# A Disulfidptosis-Related Gene Signature Associated with Prognosis and Immune Cell Infiltration in Osteosarcoma

**DOI:** 10.3390/bioengineering10101121

**Published:** 2023-09-25

**Authors:** Pengyu Chen, Jingnan Shen

**Affiliations:** Department of Musculoskeletal Oncology, The First Affiliated Hospital of Sun Yat-sen University, Guangzhou 510080, China

**Keywords:** osteosarcoma, prognosis, disulfidptosis, tumor microenvironment, bioinformatics analysis

## Abstract

Osteosarcoma (OS) stands as a leading aggressive bone malignancy that primarily affects children and adolescents worldwide. A recently identified form of programmed cell death, termed Disulfidptosis, may have implications for cancer progression. Yet, its role in OS remains elusive. To elucidate this, we undertook a thorough examination of Disulfidptosis-related genes (DRGs) within OS. This involved parsing expression data, clinical attributes, and survival metrics from the TARGET and GEO databases. Our analysis unveiled a pronounced association between the expression of specific DRGs, particularly MYH9 and LRPPRC, and OS outcome. Subsequent to this, we crafted a risk model and a nomogram, both honed for precise prognostication of OS prognosis. Intriguingly, risks associated with DRGs strongly resonated with immune cell infiltration levels, myriad immune checkpoints, genes tethered to immunotherapy, and sensitivities to systematic treatments. To conclude, our study posits that DRGs, especially MYH9 and LRPPRC, hold potential as pivotal architects of the tumor immune milieu in OS. Moreover, they may offer predictive insights into treatment responses and serve as reliable prognostic markers for those diagnosed with OS.

## 1. Introduction

Osteosarcoma (OS) is a formidable malignant neoplasm that predominantly affects adolescents during periods of rapid growth. Its genesis is commonly traced to the epiphysis of long bones, and it often portends a bleak prognosis. Without intervention, OS can lead to severe disability. Alarmingly, recurrence and metastasis, especially to the lungs, are not uncommon, further complicating the prognosis [1,2]. However, there is a silver lining: innovations in both adjuvant and neoadjuvant chemotherapy have heralded better prognostic outcomes for these patients. Yet, the survival rates for those with recurrent disease or lung metastases remain stagnant and, quite frankly, dismal [3]. Earlier studies indicated that the five-year survival for OS patients with localized disease is more than 70%, while OS with lung metastases or relapsing drops to 25% [4,5]. Hence, delving deeper into the disease’s underpinnings and unveiling novel prognostic markers are of paramount importance for improving OS patient outcomes.

Liu et al., in a groundbreaking study, introduced the academic world to "disulfidptosis," an until-now uncharted form of programmed cell death [6]. This cellular demise is triggered when cells, deprived of glucose and overexpressing SLC7A11, accumulate intracellular disulfides [7]. Distinct from other cell death pathways such as ferroptosis and apoptosis, disulfidptosis hinges on the actin cytoskeleton’s susceptibility to disulfide stress. The research posits that curtailing tumor proliferation might be achievable by inducing disulfidptosis through inhibiting glucose transporters, thereby highlighting its potential utility in oncology. This mechanism, by blocking glucose uptake in neoplastic cells, diminishes NADPH production, disrupts the actin-cytoskeletal protein balance, and eventually precipitates disulfidptosis [8]. Disulfidptosis has been linked to the prognosis of several tumors, including lung adenocarcinoma [9], hepatocellular carcinoma [10], renal cell carcinoma [11], and colon adenocarcinoma [12]. Further, the immune microenvironment critically enables cancer cells to circumvent immune surveillance and is deemed a central factor influencing treatment responses in osteosarcoma [13,14]. As a result, therapies aiming to modify the immune microenvironment and utilize pre-existing immunity to eliminate osteosarcoma cells are garnering attention for their substantial clinical promise [15,16]. There is compelling evidence to suggest that disulfidptosis might have a bearing on immune cell infiltration, potentially influencing cancer evolution, immune evasion, and therapeutic outcomes, especially in conditions such as lung adenocarcinoma [17,18]. Intriguing correlations between SLC7A11 and various immune cells in cancer have also been reported, hinting at SLC7A11s role in modulating T-cell functionality in lung adenocarcinoma [19]. Despite its recent emergence, many aspects of disulfidptosis in oncology and the immune microenvironment remain unexplored.

Given these insights, our research objectives were twofold: (1) To develop a Disulfidptosis-related signature risk model based on Disulfidptosis-related genes (DRGs) for predicting OS outcomes. (2) To evaluate drug responses and identify immune characteristics within the tumor environment by comparing high-risk and low-risk groups. The results implied that understanding DRGs could significantly enhance the accuracy of OS prognostic evaluations, offering insights into tailored treatment strategies for osteosarcoma.

## 2. Materials and Methods

### 2.1. Data Acquisition

Clinical and RNA expression data for OS patients were sourced from the TARGET (https://www.cancer.gov/ccg/research/genome-sequencing/target, accessed on 27 June 2022) and GEO (https://www.ncbi.nlm.nih.gov/geo/, accessed on 3 September 2022) databases. Datasets with pertinent transcriptional expression data accompanied by essential clinical features, including survival data of the respective patients, were incorporated into the current study. After excluding data without prognostic information, 86 OS patients from TARGET and 53 from GSE21257 were designated as the training and validation cohorts, respectively. Gene sets linked to disulfidptosis were extracted from prior studies [6]. The R package “sva” was used to eliminate the batch effect of different databases [20].

### 2.2. Disulfidptosis-Associated Subtype Identification

The “ConsensusClusterPlus” package facilitated consensus clustering of the OS cohort into distinct subtypes to probe the interplay between expression profiles [21]. Optimal cluster numbers were determined via cumulative distribution function (CDF) analysis. Subsequent examinations included comparisons of expression characteristics, prognosis using principal component analysis (PCA) plots by the “factoextra” package, and Kaplan-Meier (K-M) survival analysis by the “survminer” package, respectively. Immune cell infiltration abundance across subtypes was assessed using the CIBERSORT algorithm by the “CIBERSORT” package.

### 2.3. Gene Signature and Risk Score Formulation

To pinpoint a disulfidptosis-related prognostic gene signature (DRS) for OS, initial candidate genes were screened by univariate Cox analysis using the “survival” R package. Genes with a *p* value < 0.05 were considered to significantly relate to OS prognosis. A least absolute shrinkage and selector operation (LASSO) regression analysis, via the “glmnet” package [22], further refined this selection. A gene signature and corresponding risk score system emerged based on the intersection of genes from both analyses. The “survminer” package determined the optimal risk score cut-point, facilitating patient categorization into high-risk (HR) and low-risk (LR) groups. Risk and survival state diagrams, along with the PCA plot, gauged the model’s efficacy. The 3D PCA plot is generated using the “plot3D” package.

### 2.4. Survival Prognosis Evaluation

The prognostic merit of the signature risk score was appraised using the Kaplan-Meier survival curve, as orchestrated by the “survival” and “survminer” packages. The time-dependent ROC curves, executed by the “timeROC” package [23], further scrutinized the prognostic efficacy.

### 2.5. Nomogram Model Development and Validation

The “rms” R package was used to craft nomograms forecasting 1, 3, and 5-year OS patient prognosis. Multivariate Cox regression analysis discerned independent prognostic factors, including clinical features, for inclusion. Calibration plots, sourced from 1000 bootstrap resamples, evaluated the predictive performance of the nomogram by comparing predicted and actual survival rates. Furthermore, the prognostic efficacy of the nomogram was evaluated by time-dependent ROC.

### 2.6. Functional Enrichment Analysis

Differential transcriptional expression data analysis was orchestrated using the “limma” package [24]. Genes with a *p* value < 0.05 and |log2fold change| > 1 were considered differentially expressed genes (DEGs). Gene Ontology (GO) and Kyoto Encyclopedia of Genes and Genomes (KEGG) Enrichment analyses were facilitated by the “clusterProfiler” and “enrichplot” packages [25], with further insights from GSEA and GSVA analyses [26,27]. Terms or pathways with an adjusted *p* value < 0.05 and a FDR < 0.25 were considered significantly enriched.

### 2.7. Tumor Microenvironment and Chemotherapeutic Sensitivity Examination

The ESTIMATE [28] algorithm evaluated the tumor micro-environment (TME) by determining the stromal, immune, and ESTIMATE scores. Differences in the infiltration of immune cell subsets between risk groups were probed using the CIBERSORT [29] and ssGSEA [30] algorithms. Expression variances of immune checkpoint genes, such as PDCD1, TIGIT, IDO2, LAIR1, LAG3, CD40LG, TNFSF15, CD160, etc., and chemotherapeutic sensitivities across risk groups were assessed, with the latter employing the “oncopredict” package.

### 2.8. Statistical Analyses

All analyses were executed in R (version 4.1.2) with pertinent packages. Log-rank testing was used to determine significant differences in survival analysis using the K-M method. Univariate Cox regression analysis was conducted for estimating the hazard ratio (HR) and the 95% confidence interval (CI) values for gene expression, risk scores, and clinical parameters. Categorical variables were compared using the Chi square test. For continuous variables, the student’s t test was used for comparison between the two groups conforming to the normal distribution. The Wilcox test was used for data that did not conform to a normal distribution. A *p* value < 0.05 was considered statistically significant.

## 3. Results

### 3.1. Molecular Characteristics of DRGs-Cluster Subtypes in Osteosarcoma

**Figure 1:**
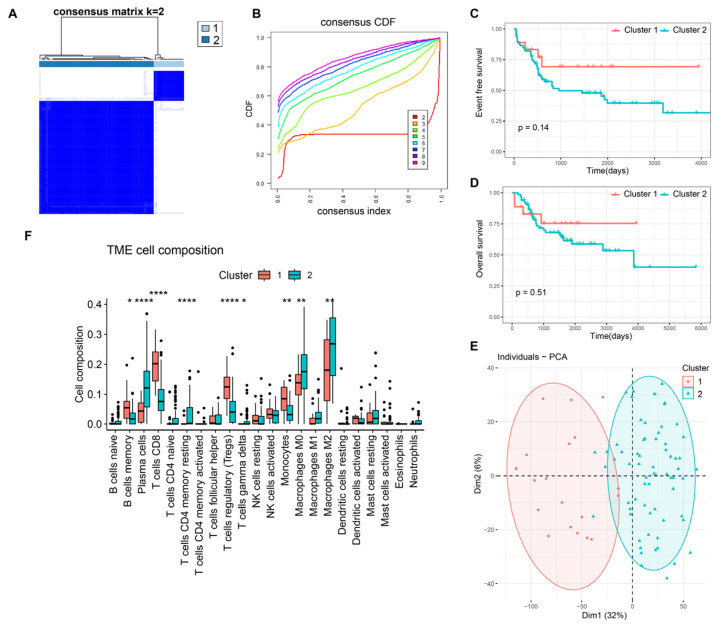
Molecular characteristics of disulfidptosis-related gene cluster subtypes in osteosarcoma. (**A**) Identification of disulfidptosis-related gene cluster subtypes. (**B**) CDF curve of consensus clustering. Event-free survival (**C**) and overall survival (**D**) analysis of osteosarcoma in gene clusters. (**E**) Principal component analysis (PCA) of gene clusters. (**F**) Immune cell infiltration assessed by the CIBERSORT algorithm in gene clusters. *, *p* < 0.05; **, *p* < 0.01; ****, *p* < 0.0001.

From the consensus clustering analysis of 12 DRGs, two gene cluster subtypes emerged: gene cluster 1 (18 samples) and gene cluster 2 (68 samples) (Figure 1A,B). Clinical outcomes indicated superior prognosis in gene cluster 2 in terms of overall and event-free survival (EFS) (Figure 1C,D), although the difference lacked statistical significance. The PCA plot revealed distinct patterns among these subtypes (Figure 1E). Moreover, disparities in immune cell infiltration between clusters were evident (Figure 1F). In Cluster 1, memory B cells, CD8-positive T cells, regulatory T cells, and monocytes show significant aggregation, while plasma cells, resting CD4-positive T cells, M0-type macrophages, and M2-type macrophages are notably suppressed compared to Cluster 2.

### 3.2. Building a Disulfidptosis-Related Prognostic Model

**Figure 2:**
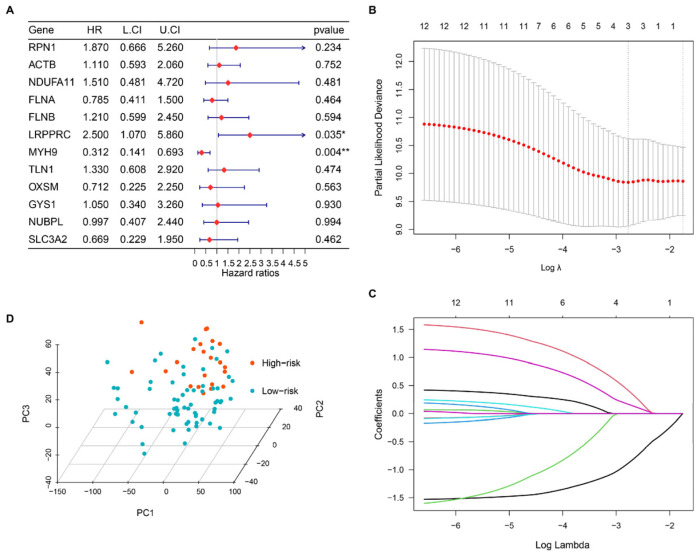
Screening for the prognostic Disulfidptosis-related Gene Signature (DRS). (**A**) A forest plot showing the results of univariate Cox regression analyses. (**B**,**C**) LASSO analysis with minimal lambda to identify prognostic variables. (**D**) 3D PCA plot of risk groups. *, *p* < 0.05; **, *p* < 0.01.

Univariate Cox regression analysis identified two pertinent DRGs from 86 OS samples in the TARGET database (training cohort): protective factor MYH9 (Hazard Ratio = 0.312, *p* = 0.004) and risk factor LRPPRC (Hazard Ratio = 2.5, *p* = 0.035) (Figure 2A). LASSO regression, which selected 3 vital genes, including MYH9, TLN1, and LRPPRC, from 12 variables, corroborated these DRGs prognostic relevance (Figure 2B,C). A risk score was computed, with patients stratified into high-risk (HR, n = 22) and low-risk (LR, n = 64) groups, respectively, using the “survminer” package. The calculation of the risk score is as follows: Risk score = (0.917 × LRPPRC expression) + −1.16 × MYH9 expression. Distinct sample characteristics among risk groups were evident in the PCA plot (Figure 2D).

**Figure 3:**
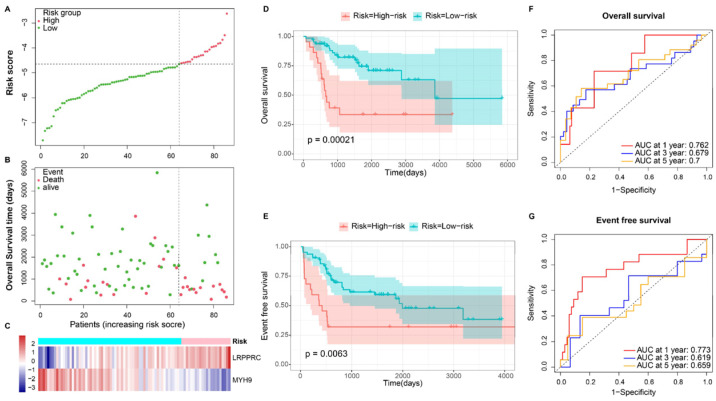
Prognosis of the DRS risk score model in the training set. (**A**,**B**) Survival status and risk score distribution between low-risk and high-risk subgroups in the training set. (**C**) Heatmap of two prognostic signature genes in different risk groups. (**D**,**E**) Kaplan-Meier curve of risk score for overall survival and event-free survival (EFS). (**F**,**G**) Time-dependent ROC curves evaluating the accuracy of the risk score for predicting overall survival and EFS in 1, 3, and 5 years, respectively.

**Figure 4:**
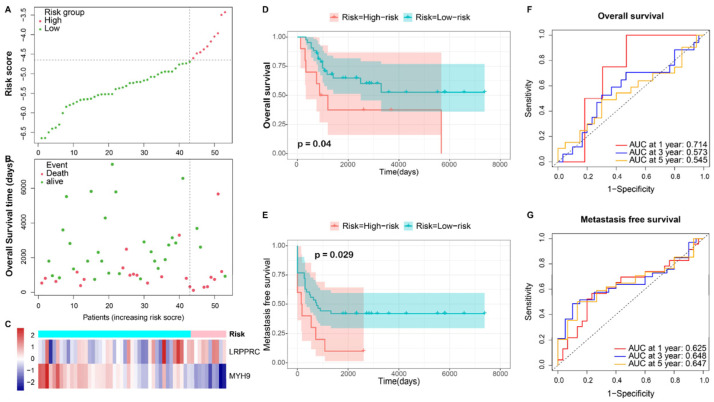
Prognosis of the DRS risk score model in the validation set. (**A**,**B**) Survival status and risk score distribution between low-risk and high-risk subgroups in the validation set. (**C**) Heatmap of two prognostic signature genes in different risk groups. (**D**,**E**) K-M curve of risk score for overall survival and metastasis-free survival (MFS). (**F**,**G**) Time-dependent ROC curves evaluate the accuracy of the risk score for predicting overall survival and MFS in 1, 3, and 5 years, respectively.

Distinct patterns between LR and HR were evident in the risk score, survival status, and heatmap of prognostic DRGs (Figure 3A–C). The KM survival analysis indicated a poorer prognosis, including overall survival (*p* = 0.00021) and EFS (*p* = 0.0063) for the HR group (Figure 3D,E). The time-dependent ROC curve affirmed the model’s robustness, with 1, 3, and 5-year Area Under Curve (AUC) values of 0.762, 0.679, and 0.700 for predicting overall survival and 0.773, 0.619, and 0.659 for predicting EFS, respectively (Figure 3F,G). Similar findings were replicated in the validation cohort, where distinct patterns were observed between different risk groups (Figure 4A–C). KM survival analysis highlighted significant differences in overall survival (*p* = 0.04) (Figure 4C) and metastasis-free survival (MFS) (*p* = 0.029) (Figure 4D) between the risk groups. The 1, 3, and 5-year AUC values were 0.714, 0.573, and 0.545 for overall survival, and 0.625, 0.648, and 0.647 for MFS, respectively (Figure 4F,G).

### 3.3. Independence Validation of the Prognostic Model

**Figure 5:**
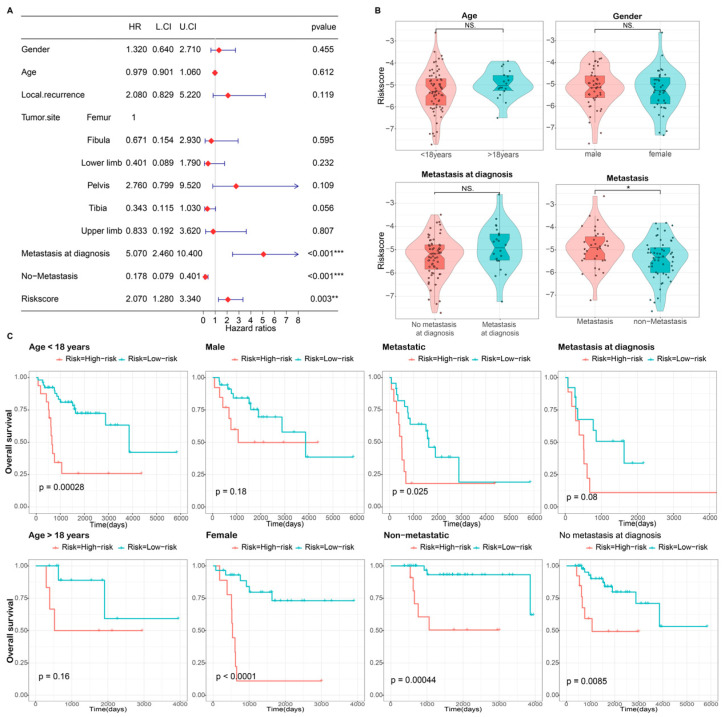
Association between the DRS risk score and clinical variables. (**A**) Univariate Cox regression analysis of clinical variables and risk score, represented by a forest plot. (**B**) Correlation analysis between clinical characteristics and risk score. (**C**) Clinical prognosis analysis of osteosarcoma with low-risk and high-risk subgroups in different clinical subgroups. L.CI: lower confidence interval; U.CI: upper confidence interval. NS, not significant; **, *p* < 0.01; ***, *p* < 0.001.

In evaluating the prognostic significance of DRG signatures in clinical contexts, we employed univariate Cox regression models. Our aim was to ascertain if the risk score, alongside variables including gender, age, metastatic presence, local recurrence patterns, and primary tumor location, can function as autonomous predictors for OS patients. The results of the univariate Cox regression analysis revealed that the risk score (Hazard Ratio = 2.070, *p* = 0.003), no metastasis (Hazard Ratio = 0.178, *p* < 0.001), and metastasis status at diagnosis (Hazard Ratio =5.070, *p* < 0.001) were significantly associated with the overall survival of OS patients (Figure 5A). While age, gender, and metastasis status at diagnosis were comparably distributed between risk groups, a higher risk score was observed in metastatic patients compared to non-metastatic patients (*p* < 0.05) (Figure 5B). Additionally, the Kaplan-Meier curves demonstrated that HR patients in each subgroup exhibited a poor prognosis, although statistical significance was not observed in all cases (Figure 5C). These findings suggest that the developed risk model possesses significant independent prognostic capability in predicting the prognosis of OS.

### 3.4. Prognostic Nomogram Construction and Validation

**Figure 6:**
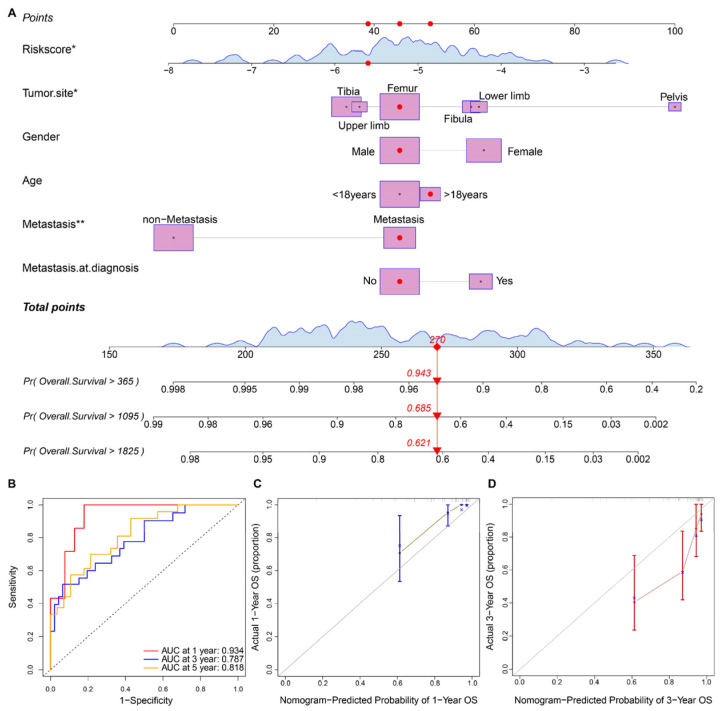
Construction of a nomogram to predict osteosarcoma survival. (**A**) Nomogram construction of the DRS risk score and clinical variables. The red dots and red line show an example of points calculating and corresponding predicted survival of the nomogram. (**B**) ROC curve analysis shows the AUC of the nomogram. (**C**,**D**) 1 year and 3 year calibration curve for overall survival prediction of the nomogram. The gray lines represent perfect fit lines between predicted and actual probabilities. OS: Overall survival. *, *p* < 0.05; **, *p* < 0.01.

To enhance the prediction of 1, 3, and 5-year survival in OS patients, we constructed a nomogram integrating the risk score with clinical attributes including gender, age, metastatic status, metastatic status at diagnosis, local recurrence status, and primary tumor site (Figure 6A). This nomogram facilitates survival prediction by assigning scores to each variable based on the patient’s specific attributes, ultimately yielding a total score that predicts survival at specific time points (1, 3, and 5-years). The performance of the nomogram was evaluated using ROC curves and calibration curves (Figure 6B–D). The 1, 3, and 5-year AUC values were 0.934, 0.787, and 0.818 for overall survival prediction. The calibration curves depicted a close alignment between the actual and optimized survival probabilities, as indicated by the solid and dashed lines, respectively, demonstrating an excellent fit of the prediction.

### 3.5. Gene Function Enrichment Analysis

**Figure 7:**
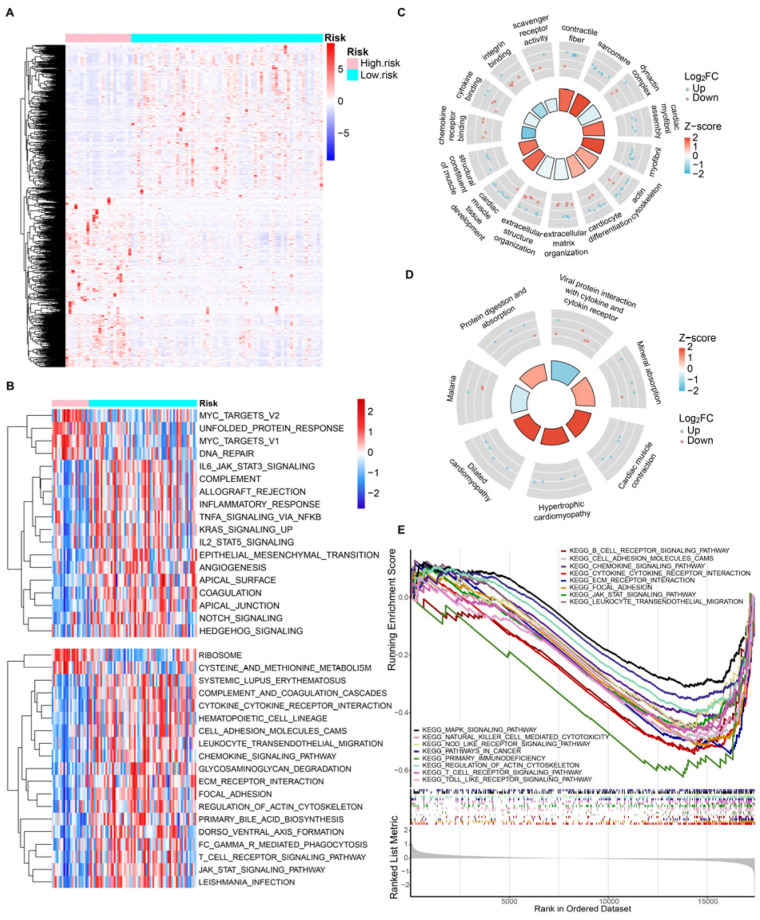
Gene function enrichment analysis of risk groups. (**A**) Heatmap of risk group-related differentially expressed genes. (**B**) GSVA analysis of hallmark and KEGG pathways between risk groups. (**C**,**D**) A circle plot of the GO and KEGG enrichment analyses of risk group-related DEGs. (**E**) GSEA analysis of KEGG pathways between risk groups.

To further explore the underlying biological processes and signaling pathways, we identified a total of 102 DEGs, including 62 upregulated genes and 40 downregulated genes, between risk groups in the TARGET OS cohort. Distinct risk group patterns emerged from the analysis of differentially expressed genes (DEGs) in osteosarcoma patients, as shown by the heatmap (Figure 7A). Further analysis using Gene Set Variation Analysis (GSVA) demonstrated that the HR samples exhibited enrichment in cancer hallmarks, including DNA repair, epithelial-mesenchymal transition, and angiogenesis, among others. Results from the KEGG pathway analysis revealed that risk-related DEGs were significantly enriched in immunity-related pathways, such as focal adhesion, cytokine-cytokine receptor interaction, and the T cell receptor signaling pathway. Additionally, cellular structure-related processes, such as regulation of the actin cytoskeleton, were also found to be enriched (Figure 7B). Figure 7B shows 18 significant hallmark pathways with an adjusted *p* value < 0.01 and 19 significant KEGG pathways with an adjusted *p* value < 0.001. To confirm these findings, Gene Set Enrichment Analysis (GSEA) was performed, which further validated the enrichment of cellular structure and immunity-related biological processes and pathways among risk-related DEGs (Figure 7C–E). The top five terms of biological processes, molecular functions, and cell contents are shown, respectively, in Figure 7C. DEGs are shown to be involved in biological processes such as extracellular matrix organization and extracellular structure organization, as well as molecular functions such as chemokine receptor binding, cytokine binding, integrin binding, etc. In Figure 7D,E, we highlight the pronounced associations these DEGs have with KEGG pathways. These encompass B cell receptor signaling, chemokine signaling, leukocyte transendothelial migration, natural killer cell-mediated cytotoxicity, T cell receptor signaling, etc. These results serve as evidence that these DEGs are intrinsically linked with the immune microenvironment in osteosarcoma samples. Such connections may offer insights into potential mechanisms that forecast the prognosis for osteosarcoma patients.

### 3.6. Tumor Micro-Environment Characteristics in Risk Groups

**Figure 8:**
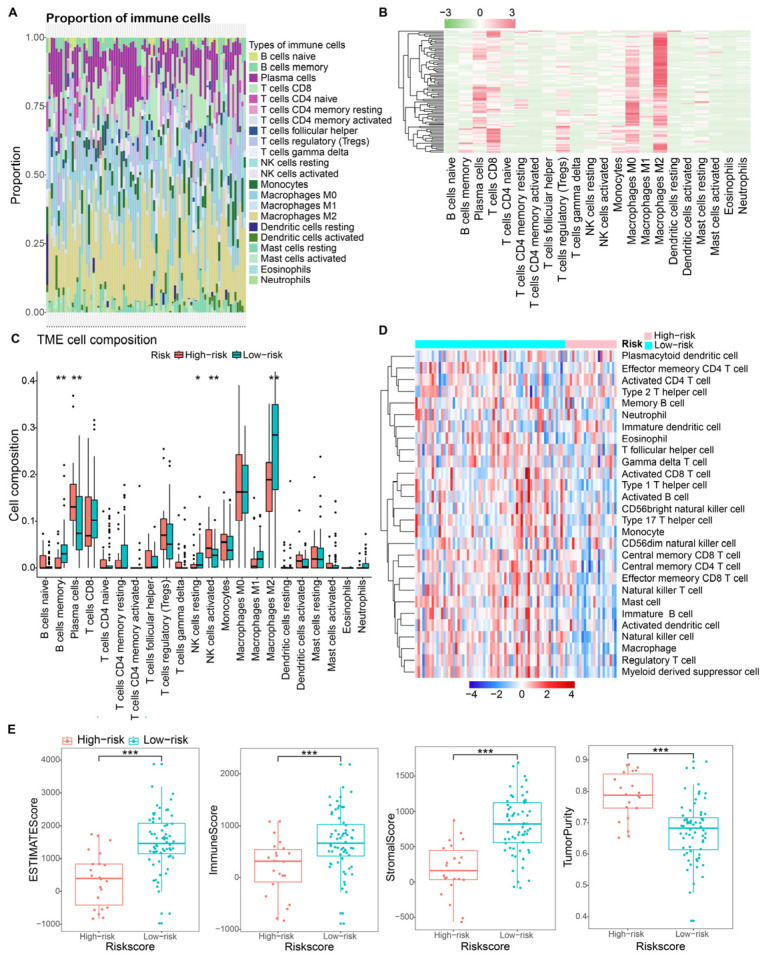
Immune landscape between DRS risk groups. (**A**,**B**) Immune cell landscape of osteosarcoma samples; (**C**) Tumor microenvironment (TME) cell infiltration among high-risk and low-risk groups. (**D**) Heatmap depicting the enriching level of 28 immune-related cells evaluated by the ssGSEA algorithm. (**E**) Immune scores, stromal scores, ESTIMATE scores, and tumor purity among high- and low-risk groups. *, *p* < 0.05; **, *p* < 0.01; ***, *p* < 0.001.

To elucidate the association between DRG-related risk and immune characteristics, we employed the CIBERSORT algorithm to compute the enrichment scores of a diverse range of immune cell populations. Figure 8A,B depict the landscape of immune cell infiltration, emphasizing the prominent presence of CD8 T cells, regulatory T cells, Macrophages M0, Macrophages M2, and plasma cells within the microenvironment of osteosarcoma. Subsequently, the CIBERSORT algorithm highlighted varied immune cell infiltration between risk groups. The results demonstrated a significant disparity in the abundance of memory B cells, resting NK cells, activated NK cells, and Macrophages M2 between HR and LR (Figure 8C). Notably, Figure 8C illustrates a higher abundance of activated NK cells in HR osteosarcoma samples, whereas memory B cells, resting NK cells, and Macrophages M2 were found to be down-regulated. ssGSEA further demarcated distinct immune cell infiltration patterns between risk groups through heatmap visualization (Figure 8D). Additionally, we observed significant differences in HR patients compared to LR patients, with lower calculated immune scores (*p* < 0.001), stromal scores (*p* < 0.001), ESTIMATE scores (*p* < 0.001), and higher tumor purity indices (*p* < 0.001). (Figure 8E). 

### 3.7. Immune Checkpoint and Drug Sensitivity Analysis

**Figure 9:**
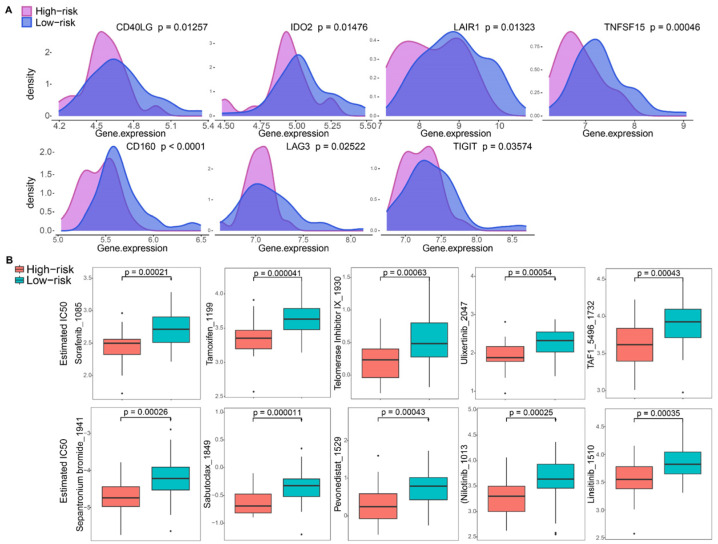
Evaluation of immune checkpoints and chemotherapeutic drug sensitivities in high-risk and low-risk groups. (**A**) Expression of immune checkpoint signatures among high-risk and low-risk groups. (**B**) Chemotherapeutic drug sensitivity analysis of representative drugs in different risk groups.

To assess immune checkpoint inhibitors (ICIs) for OS, the association between risk groups and prevalent ICI targets was examined, revealing differences in the expression of checkpoint-related genes. The results demonstrated a significant difference in the expression of immune checkpoint-related genes between the risk groups, including CD40LG, IDO2, LAIR1, TNFSF15, CD160, LAG3, and TIGIT (Figure 9A). All ICIs mentioned above were found down-regulated in HR samples. Systematic treatment, including chemotherapy and targeted therapy, has gained increasing prospects in OS. Consequently, an examination of the sensitivity of different risk score groups to specific drugs was performed, aiming to identify potential treatment options guided by the identified risk groups. Data from GDSC2, encompassing pharmacological information, revealed that the effects of 74 drugs (*p* < 0.05) were associated with the risk groups. In particular, the IC50 values for representative drugs, including sorafenib, tamoxifen, telomerase inhibitor IX, ulixertinib, TAF1_5496, sepantronium bromide, sabutoclax, pevonedistat, nilotinib, and linsitinib, were presented in Figure 9B, demonstrating a statistically significant lower IC50 in HR samples with *p* < 0.001.

## 4. Discussion

Osteosarcoma (OS) stands as one of the most aggressive bone cancers, predominantly affecting children and adolescents globally. Despite the advent of diverse treatments—surgery, radiotherapy, chemotherapy, and neoadjuvant chemotherapy—the five-year survival rate for OS has seen little improvement over the past three decades. This underscores an urgent need for personalized diagnostic and therapeutic strategies. [31,32]. Cell death is integral to tumor proliferation regulation [33], and its interplay with cancer cell metabolism has been the subject of extensive research [34]. Elevated SLC7A11 expression has been linked to apoptosis in glucose-starved cancer cells [35]. Conversely, inhibiting disulfide accumulation has been shown to prevent cell death in the presence of high SLC7A11 expression [36]. Such insights underscore the intricate relationship between disulfide stress and cell death. The concept of disulfidptosis, introduced by Liu et al. [6], offers a fresh perspective on the role of disulfides and aberrant glucose metabolism in cell death. SLC7A11 high-expression cells under glucose starvation conditions accumulate excessive intracellular disulfides, resulting in disulfide stress, which leads to disulfidptosis. By hindering glucose uptake in cancer cells, metabolic treatment with glucose transporter inhibitors induces disulfidptosis and hampers the growth of neoplasms akin to renal cell carcinoma. This inhibition of glucose uptake dilutes NADPH production, elevates the NADP+/NADPH ratio, and promotes the irregular formation of actin-cytoskeletal protein disulfide bonds and devolution of the F-action network, ultimately instigating disulfidptosis [8]. However, a detailed grasp of disulfidptosis-related genes (DRGs) in cancer processes has yet to be achieved. Consequently, additional research into disulfidptosis is imperative to enrich our comprehension of its possible utility in cancer treatment. Besides, disulfidptosis has been found to be associated with tumor prognosis in several cancer types, such as lung adenocarcinoma [9], hepatocellular carcinoma [10], renal cell carcinoma [11], colon adenocarcinoma [12], etc. Our data demonstrate the accuracy of risk stratification based on disulfidptosis-related factors in predicting the prognosis of osteosarcoma, highlighting the clinical significance of these genes in osteosarcoma prognosis. Previous studies have reported that disrupted SLC7A11 expression influences the survival and migration abilities of osteosarcoma cells [37,38]. Targeting SLC7A11 in vivo has been shown to impede tumor growth in osteosarcoma [39]. However, the precise role bridging disulfidptosis and metabolism in OS or the broader field of oncology demands further investigation. In this study, we meticulously analyzed 12 previously identified DRGs in osteosarcoma, and identified two clusters with distinct patterns using NMF consensus clustering. We have identified noteworthy trends in principal components, patient prognosis (represented by overall survival and event-free survival), and immune cell infiltration status, even though some of these trends are not statistically significant. To further explore and enhance the predictive capability of DRGs, we identified two DRGs (MYH9 and LRPPRC) by LASSO and univariate Cox regression analyses to build a disulfidptosis-related prognostic gene signature (DRS) (Figure 2).

Myosin heavy chain 9 (MYH9) has a documented role in various diseases, including hereditary diseases, hematologic diseases, and inflammatory conditions [40,41,42,43,44]. Recent research has revealed that MYH9 also plays a role in driving tumor cell migration and invasion. For instance, Katono et al. [45] discovered that non-small cell lung cancer tissue with positive MYH9 expression exhibited a higher incidence of intra-tumor vascular invasion and tumor node metastasis, as well as a poorer prognosis for patients. Zhou et al. [46] found that mRNA expression levels and protein levels of MYH9 were significantly elevated in osteosarcoma tissues compared to benign osteochondroma tissues. High expression of MYH9 in osteosarcoma tissues showed a clear association with a higher Enneking classification (III classification) and the presence of lung metastasis. Downregulation of MYH9 expression effectively impaired the migration and invasion capabilities of osteosarcoma cell line SAOS2 cells. Tang et al. [47] have provided evidence indicating that MYH9 plays a crucial role in the regulation of immune response and cell proliferation in lung adenocarcinoma. They have shown that MYH9 controls the secretion of significant immune-related cytokines, highlighting its involvement in immunological and proliferative processes in tumor cells. These results imply that further exploration of the prognostic and migratory implications of MYH9 in osteosarcoma is warranted.

Leucine-rich pentatricopeptide repeat-containing protein (LRPPRC) has recently emerged as a novel N6-methyladenosine (m6A) modification reader with potential implications for tumor progression. Prior investigations underscored that inhibiting LRPPRC activity curtailed tumor expansion in murine models. Notably, this inhibition appears to post-transcriptionally amplify PD-L1, perhaps through an m6A-dependent process, thus enhancing the mRNA stability of PD-L1. Such findings illuminate a fresh avenue by which m6A regulators might facilitate immunosuppression in hepatocellular carcinoma [48]. Further delving into its molecular pathways, LRPPRC supports both Yap-P27-driven cell polyploidy and P62-HDAC6-guided autophagy maturation. These actions collectively work to minimize genome instability, subsequently deterring the advancement of hepatocellular carcinoma [49]. In another intriguing interplay, LRPPRCs interaction with the long non-coding RNA (lncRNA) SNHG17 stabilizes the c-Myc protein. This molecular synergy expedites the G1/S transition and bolsters cellular proliferation in hepatocellular carcinoma [50]. In terms of immune modulation, LRPPRC generally presents an inverse relationship with the majority of tumor-infiltrating immune cells [51]. An upward trend between LRPPRC and PD-L1 expression has been observed [52]. Heightened LRPPRC expression correlates with diminished populations of T cells, cytotoxic cells, and dendritic cells and compromised cytolytic function [53]. Additionally, LRPPRC may potentially impact the MHC molecules HLA-B and HLA-DOA in periodontitis [54]. In the present study, we identified LRPPRC as a risk factor for OS prognosis (Hazard Ratio = 2.5, *p* = 0.035) through Cox analysis. In summary, we assume that LRPPRC may potentially promote the progression of osteosarcoma by exerting immunosuppressive effects. Nevertheless, the intricate pathways and effects of LRPPRC, along with associated DRGs, on osteosarcoma progression and immune cell infiltration remain enigmatic and warrant deeper investigation.

In further analysis of clinical features, we recognized metastatic status as a critical influencer for OS outcomes (Figure 5A), especially in combination with the disulfidptosis-related prognostic gene signature (DRS). To further refine prognostic insights, we designed a comprehensive nomogram integrating clinical features including gender, age, metastatic status, local recurrence status, and primary tumor site, which showed promising predictive accuracy (Figure 6). The performance of the nomogram in predicting prognosis was effectively demonstrated by time ROC curves at 1, 3, and 5-years along with calibration plots showing satisfying fit of predicted and observed lines, thereby further substantiating the predictive capability of the prognostic model.

The tumor microenvironment’s (TME) immune cell infiltration significantly dictates tumor traits, such as malignancy and metastatic tendencies [55,56]. Recent studies have suggested a close association between disulfidptosis and immune infiltration, with high disulfidptosis subtypes exhibiting higher immune scores [6]. In this study, we found a strong correlation between the DRS and the immunological characteristics of OS. We employed an array of methods—CIBERSORT; ESTIMATE; and ssGSEA—to gain a holistic view of the immune cell composition in osteosarcoma’s TME. Our findings revealed that higher levels of activated natural killer (NK) cells, along with lower levels of memory B cells, resting NK cells, and macrophages M2, were associated with a high DRS-related risk score and an unfavorable prognosis in OS patients (Figure 8C).

Tumor-associated macrophages (TAMs) typically exhibit the M2-pro-tumoral phenotype. Typically, the M1 macrophage phenotype is involved in inflammation, inversely affecting metastasis, while the M2 phenotype plays roles in wound healing and immune regulation, promoting metastasis [57]. The correlation of M2 TAMs with tumor aggressiveness and unfavorable prognosis is well established in various cancer types [58]. In osteosarcoma’s immune microenvironment, TAMs dominate, constituting approximately 50% of the total tumor volume [59]. However, their role in osteosarcoma (OS) remains ambiguous [31]. While many studies suggest a pro-tumoral role consistent with other tumor types, some associate TAMs with a favorable prognosis, suggesting anti-tumor activity. Xiao et al. demonstrated that in a human OS xenograft mouse model, the tumor-recruited macrophages predominantly exhibit the M2 subtype, and their elimination curtails tumor growth [60]. Similarly, Shao et al. found that M2 macrophages bolster OS initiation and progression, as evidenced by orthotopic co-injections in mouse tibia using the K7M2 OS cell line alongside RAW264.7 macrophagic cells [61]. Contraintuitively, some research implies that the M2 phenotype might exhibit anti-tumor and anti-metastatic properties in osteosarcoma [62,63]. Cao et al. observed that osteosarcoma cases with diminished M2 macrophages presented a notably poorer survival rate [64], a finding consistent with our study. Previous evidence has demonstrated that natural killer T cells are linked to a poor prognosis in OS patients, similar to our results [65]. Clinical studies have also revealed significantly reduced levels of natural killer T cells in the peripheral blood of OS patients compared to healthy individuals [66]. Additionally, myeloid cells, including macrophages, microglia, myeloid-derived suppressor cells, dendritic cells, and neutrophils, have been identified as the primary infiltrating immune cell populations within the TME [67]. These myeloid cells actively regulate immune responses and therapeutic outcomes [68]. Our findings revealed a dynamic interplay between DRS, immune cell infiltration, and OS prognosis, suggesting that these genes may have a profound role in osteosarcoma’s metastasis and progression.

The tumor immune environment is intricate, encompassing not just immune cells but also various factors such as immune checkpoints, regulatory cells, inflammatory cytokines, and the broader tumor microenvironment, all of which can influence immune function. Immune checkpoint inhibitors (ICIs) have emerged as potent anti-cancer agents and are now deployed as first-line treatments for several solid tumors [69]. Prior research has shown that osteosarcoma (OS) with elevated PD-L1 expression correlates with a higher metastatic risk and poorer survival. This suggests that exploring ICIs for OS treatment might be a logical strategy [70]. However, the clinical effectiveness of checkpoint inhibitors, alone or in combinations, remains limited in OS, as evidenced by several trials. Additionally, most studies indicate that high PD-L1 expression does not consistently predict immunotherapy responsiveness in OS. There is a pressing need for alternative immune biomarkers and treatment strategies for OS. In our study, we observed a significant increase in the expression of various ICIs in the low-risk (LR) group, encompassing CD40LG, IDO2, LAIR1, TNFSF15, CD160, LAG3, and TIGIT (Figure 9A). This suggests that such patients may have enhanced immunoreactivity and could potentially benefit more from ICI treatments [71]. Nonetheless, further studies are crucial to determine if targeting these checkpoints offers a promising avenue for OS therapy.

Additionally, the risk categorization based on the 2-DRGs signature revealed differentiated drug sensitivities. Drugs such as Sorafenib, Tamoxifen, Telomerase Inhibitor IX, Ulixertinib, TAF1_5496, Sepantronium Bromide, Sabutoclax, Pevonedistat, Nilotinib, and Linsitinib emerged as potential candidates better suited for the high-risk (HR) group (as depicted in Figure 9B). The lower IC50 values for these drugs in the HR group suggest their prospective efficacy for patients within this classification. Such insights from our research may assist in refining drug selection for patients based on their respective risk profiles for upcoming therapeutic strategies.

This study presents several limitations: (1) The expression data employed originated from the TARGET and GSE21257 datasets. These are somewhat antiquated when juxtaposed with contemporary techniques, potentially affecting compatibility with results from fresh analyses. This disparity could limit the further validation and practicality of our findings. It is anticipated that future analyses using recent, high-quality OS cohorts will yield a more definitive and precise prognostic model. (2) Our study relies on a dataset with a confined sample size. Expanding the scope of subsequent studies will not only solidify the evidence but also enhance the predictive reliability of our model. (3) Our analytical approach was predominantly bioinformatics-based, facilitating the construction of the prognostic model and the exploration of potential DRG-OS associations. However, for empirical corroboration, it is paramount to execute biological experiments. Such endeavors in our next study would affirm the DRGs expression patterns and unearth the molecular pathways dictating their influence on OS prognosis and immunological profiles; (4) The exact mechanisms through which DRGs modulate the immune environment and drug sensitivity remain nebulous. Comprehensive further research is essential to unpack the intricate interplay between these DRGs and OS pathogenesis, with the goal of identifying potential therapeutic intervention points.

In conclusion, our study introduces and validates a unique prognostic signature based on two DRGs (MYH9 and LRPPRC) for osteosarcoma. The association between this signature and patient survival is profound, and this study sheds light on the intricate dance of DRGs and immune cells within the TME. Moreover, the current disulfidptosis-related signature may encompass a latent connection with the drug sensitivities of OS. This opens avenues for tailoring immunotherapeutic and pharmacotherapeutic strategies for specific OS patient cohorts. However, the nexus between disulfidptosis and osteosarcoma requires more extensive research, aiming ultimately to revolutionize personalized treatment paradigms for OS patients.

## Data Availability

The datasets presented in this study can be found in online repositories. The names of the repository/repositories and accession numbers can be found in the article.
